# Evaluation of Factors Affecting the Health-Related Quality of Life in End-Stage Renal Disease Patients Receiving Hemodialysis Treatment in Northern Cyprus

**DOI:** 10.7759/cureus.67893

**Published:** 2024-08-27

**Authors:** Moomen Hassan, Ahmed N Canatan, Gizem Çakır, Ege Pastırmacıoğlu, Söğüt Yorgancı, Özge Cumaoğulları, Nimet İlke Akçay, Düriye Deren Oygar

**Affiliations:** 1 Faculty of Medicine, Marmara University, Istanbul, TUR; 2 Faculty of Medicine, Eastern Mediterranean University, Gazimağusa, CYP; 3 Nephrology, Burhan Nalbantoğlu State Hospital, Lefkoşa, CYP

**Keywords:** renal replacement therapy (rrt), health-related quality of life (hrqol), end-stage renal disease (esrd), northern cyprus, kdqol-36, hemodialysis

## Abstract

Introduction

Health-related quality of life (HR-QoL) is recognized as an important predictor of mortality and morbidity in end-stage renal disease (ESRD) patients receiving hemodialysis (HD). The goal of this study was to obtain HR-QoL scores using the Kidney Disease and Quality of Life questionnaire (KDQOL-36^TM^) (RAND Corporation, Santa Monica, CA) and to assess the factors affecting HR-QoL in ESRD patients receiving HD.

Methods

A multicenter cross-sectional study was performed using the KDQOL-36^TM^ for the assessment of HR-QoL of ESRD patients receiving HD in Northern Cypriot state hospitals. Alongside KDQOL-36^TM^ scores for assessing HR-QoL, sociodemographic as well as relevant laboratory data were collected. Spearman Correlations and multiple linear regression analyses using the 'stepwise' method were carried out to identify predictors of HR-QoL. Nonparametric tests were used to determine significantly (p<0.05) associated variables. Ethical approvals were received from the Northern Cypriot Ministry of Health and Eastern Mediterranean University Research and Publication Ethics Boards.

Results

One hundred and thirty-eight participants were recruited in this study, corresponding to 85.1% of the total study population. Participants had a mean age of 66.49 ± 13.35. 65.2% of the participants were males. Participants had low scores on most dimensions of quality of life, namely, Burden of Disease, Physical Component Summary (PCS), and Mental Component Summary (MCS) subscales. Particularly, females, unemployed patients, and patients with more comorbidities had significantly lower scores (p=0.003, p<0.001, and p=0.005, respectively). Spearman correlation analyses revealed multiple significant moderate correlations between sociodemographic data, laboratory variables, and scores. Furthermore, multiple linear regression analysis identified gender (p=0.006), total number of comorbidities (p=0.005), age (p<0.001), and patient care (p=0.019) as significant predictors of KDQOL-36^TM ^scores.

Conclusion

This study has shown that the quality of life of hemodialysis patients was highly impaired. Gender, current employment status, and presence of comorbidities were all significant independent factors affecting HR-QoL mean scores. Lastly, further studies regarding the implementation of routine HR-QoL surveillance and targeted interventions are required to better understand their potential therapeutic benefits.

## Introduction

Health-Related Quality of Life (HR-QoL) is a crucial concept that is concerned specifically with health aspects while also accounting for general Quality of Life (QoL) components. The Centers for Disease Control (CDC) has defined HR-QoL as “an individual’s or group’s perceived physical and mental health over time” [1]. Evaluation of the HR-QoL is essential in improving effective patient care. Studies have shown discrepancies between the patients’ self-perception of their health status and healthcare workers’ perception of patients’ condition. Thus, it is recommended that patients should evaluate their own HR-QoL [2].

Chronic Kidney Disease (CKD) is a worldwide public health burden with high costs to healthcare systems and adverse outcomes. In stage 5 of CKD, also named End Stage Renal Disease (ESRD), Renal Replacement Therapy (RRT) is initiated to optimize kidney function [3]. There are three different RRT modalities for ESRD patients: hemodialysis (HD), peritoneal dialysis, and kidney transplantation [4]. The majority (91.1%) of the ESRD patients in the Turkish Republic of Northern Cyprus (TRNC) are receiving HD treatment [5].

A wide range of different tools were developed to measure QoL. Some of these measurement tools are generic, such as the 36-Item Short Form Health Survey (SF-36) and the World Health Organization Quality of Life - Brief (WHOQOL-BREF). The Kidney Disease Quality of Life (KD-QoL) survey, developed by the Kidney Disease Quality of Life Working Group, is a well-known example of a disease-specific measure of HR-QoL [6].

The National Kidney Foundation states that Physical and Mental Component Summary Scores of SF-36 are important predictors of mortality and morbidity in ESRD patients receiving HD treatment. One point increase in either of the Physical or Mental Component Summary Scores decreases the relative risk of mortality by 2% and hospitalization by 1% [7]. Moreover, HR-QoL is required by the Center for Medicare and Medicaid Services to be measured annually at all dialysis units in the care of ESRD patients.

Assessment of HR-QoL may inform healthcare workers about the effectiveness of a patient's current treatment. Additionally, it is essential in improving patients’ care and needs, monitoring disease progression, and identifying the subset of patients who are at increased risk and may benefit from targeted interventions [8,9].

The present study aims to obtain the result scores of ESRD patients undergoing HD by using Kidney Disease Quality of Life-36 instrument (KDQOL-36) and to assess the potential correlations among demographic characteristics, medical factors, and QoL in these patients.

## Materials and methods

Study group

This is a cross-sectional study and the participants of this study were all end stage renal disease (ESRD) patients on routine hemodialysis treatment in state hospitals of Northern Cyprus. ESRD patients younger than 18 years old, with acute kidney disease, receiving peritoneal dialysis, having cognitive impairment, dementia, or psychosis, and receiving hemodialysis for less than three months were not included in the study. After excluding 23 patients from the total population, the new total number of participants was reduced to 162.

Study tools

Information was gathered from participants by using the Turkish version of the “Kidney Disease and Quality of Life Instrument (KDQOL-36^TM^)” questionnaire with permission from the RAND Corporation (Copyright^©^ the RAND Corporation, Santa Monica, CA). In a study titled “Translation, Cultural Adaptation, Initial Reliability, and Validation of the Kidney Disease and Quality of Life - Short Form KDQOL-SF 1.3^TM^ in Turkey,” it was stated that the results demonstrated high reliability and validity of the questionnaire for Turkish patients on dialysis [10]. Notably, all questions of KDQOL-36^TM^ are directly derived from the longer version, KDQOL-SF 1.3^TM^. 

**Figure 1:**
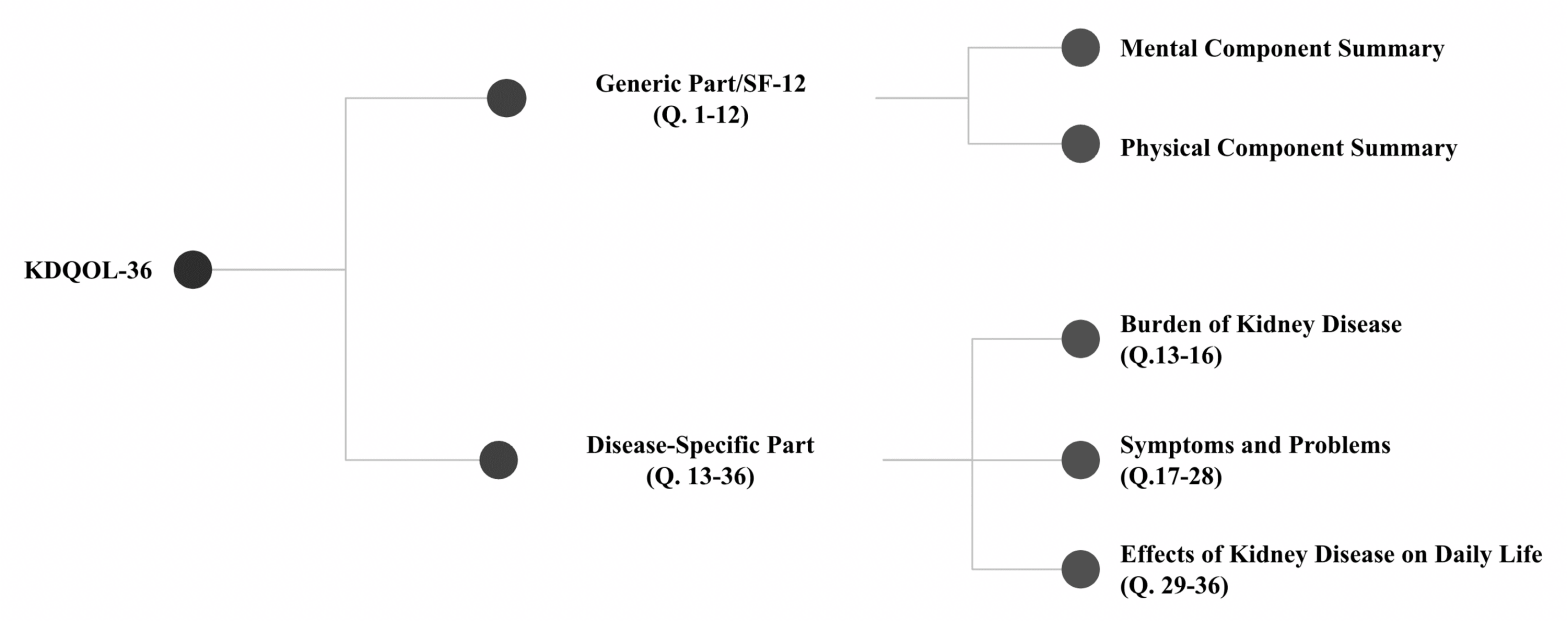
Parts and subscales of KDQOL-36 questionnaire survey Kidney Disease and Quality of Life Instrument (KDQOL-36^TM^) (RAND Corporation, Santa Monica, CA).

KDQOL-36^TM^ is composed of 36 questions with two parts and five subscales demonstrated in Figure 1. The first part is a generic part called SF-12 making up the first 12 questions. SF-12 has two subscales, namely, Mental Component Summary (MCS) and Physical Component Summary (PCS). SF-12 has norm-based scores of 50 with a standard deviation of 10. Scores less than 40 indicate a worse perception of the patient's own health. The second part of the tool is the Kidney Disease-Specific section consisting of three disease-specific subscales, namely, the Burden of Kidney Disease subscale (Questions 13-16), Symptoms and Problems subscale (Questions 17-28), and Effects of Kidney Disease on Daily Life subscale (Questions 29-36). This part has scores from 0-100, “0” being the worst and “100” being the best perception of one's own health by participants. In general, lower scores indicate worse health-related quality of life (HR-QoL). Additionally, demographic data and relevant routine lab results were collected from each participant.

Procedure 

The data was collected using self-administered questionnaires during the first quarter of 2018 at the three state hospitals in Northern Cyprus with hemodialysis. Complete blood count and biochemistry lab results were obtained from hospital archives.

Data was analyzed using SPSS Statistics for Windows, Version 25.0. (IBM Corp., Armonk, NY) and was reported with 95% confidence intervals. Non-parametric Mann Whitney U and Kruskal Wallis tests were conducted for comparative analyses. Spearman Correlations were done between quantitative data (lab parameters, months since started hemodialysis, age) and scores. Multiple Linear regression analysis was performed using the Stepwise method. Differences were considered statistically significant at p < 0.05. 

Ethics

This study was approved by the Eastern Mediterranean University Medical School Ethics Committee (Protocol number: ETK00-2019-0013), as well as the Ministry of Health (YTK.1.01) prior to data collection. The study was in adherence to the principles of the Declaration of Helsinki.

## Results

Sociodemographic and laboratory parameters

Out of the total ESRD population (n=162) receiving hemodialysis in the state hospitals of Northern Cyprus, 85.19% (n=138) participated in this study. Participants had a mean age of 66.49 ± 13.35 years. 65.22% (n=90) were males. Most participants had only finished primary education (42.75%; n=59) and were retired (64.49%; n=89). The majority had caregivers (92.75%; n=128), and for most participants the caregivers were their spouses (51.45%; n=71). Most participants were receiving hemodialysis three times a week (88.41%; n=122), and every session lasted 3-4 hours (99.28%; n=137). 75.36% (n=104) of participants did not have to wait before their dialysis sessions. Sociodemographic characteristics as well as relevant laboratory parameters of participants are listed in Tables 1, 2, respectively.

**Table 1:** Sociodemographic characteristics of the study participants

Variable	Frequency (N)	Percentage (%)
Gender		
Male	90	65.20%
Female	48	34.80%
Age Groups (years)		
≤ 65	55	39.86%
>65	83	60.14%
Marital Status		
Married	105	76.09%
Singe	9	6.52%
Widowed	24	17.39%
Education Level		
No education	10	7.25%
Primary education	59	42.75%
Secondary Education	54	39.13%
Tertiary Education	15	10.87%
Occupational status		
Unemployed	40	28.99%
Employed	9	6.52%
Retired Preferred	58	42.03%
Retired Forced	31	22.46%
Caregiver		
Spouse	71	51.45%
Son/Daughter	36	26.09%
Sibling	8	5.80%
None	10	7.25%
Others	13	9.42%

**Table 2:** Relevant laboratory parameters of the study participants

Variable	Frequency (N)	Percentage (%)
Alkaline Phosphatase (IU/L)		
Low (<47)	3	2.17%
Normal (47-147)	113	81.88%
High (>147)	19	13.77%
Missing values	3	2.17%
Parathyroid hormone (pg/mL)		
Low (<10)	3	2.17%
Normal (10-55)	2	1.45%
High (>55)	116	84.06%
Missing values	17	12.32%
Albumin (g/dL)		
Low (<3.5)	7	5.07%
Normal (3.5-5.5)	129	93.48%
High (>5.5)	0	0.00%
Missing values	2	1.45%
Hemoglobin (g/dL)		
Low (<11.6 for female, <13.2 for male)	89	64.49%
Normal (11.6-15 for female, 13.2-16.6 for male)	48	34.78%
High (>15 for female, >16.6 for male)	0	0.00%
Missing values	1	0.72%
Hematocrit (%)		
Low (<35.5 for female, <38.3 for male)	70	50.72%
Normal (35.5-44.9 for female, 38.3-48.6 for male)	66	47.83%
High (>44.9 for female, >48.6 for male)	2	1.45%
Missing values	0	0.00%
Mean Corpuscular Volume (fL)		
Low (<80)	25	18.12%
Normal (80-100)	100	72.46%
High (>100)	13	9.42%
Missing values	0	0.00%
Platelets (× 10^9^/L)		
Low (<150)	21	15.22%
Normal (150-400)	114	82.61%
High (>400)	2	1.45%
Missing values	1	0.72%

Most of the participants had at least one comorbidity (78.26%; n=108). The most common comorbidities were hypertension (53.62%; n=74), diabetes mellitus (37.68%; n=52), and diseases of the cardiovascular system (25.36%; n=35). Distribution of comorbidities of participants is summarized in Table 3.

**Table 3:** Characteristics of comorbidities among participants

Variables		
The number of comorbidities a patient has	Frequency (N)	Percentage (%)
Zero	30	21.74%
One	44	31.88%
Two	30	21.74%
Three	23	16.67%
Four	9	6.52%
Five	2	1.45%
Distribution of different comorbidities among participants	Frequency (N)	Percentage (%)
Hypertension	74	53.62%
Diabetes Mellitus	52	37.68%
Diseases of the Cardiovascular System	35	25.36%
Diseases of the Musculoskeletal System	13	9.42%
Diseases of the Nervous System	9	6.52%
Different cancer types	7	5.07%
Diseases of the Gastrointestinal System	7	5.07%
Diseases of the Eyes	5	3.62%
Hepatitis B or C	5	3.62%
Diseases of the Blood	5	3.62%
Metabolic Diseases	4	2.90%
Diseases of the Respiratory System	3	2.17%

KDQOL-36™ scores

**Figure 2:**
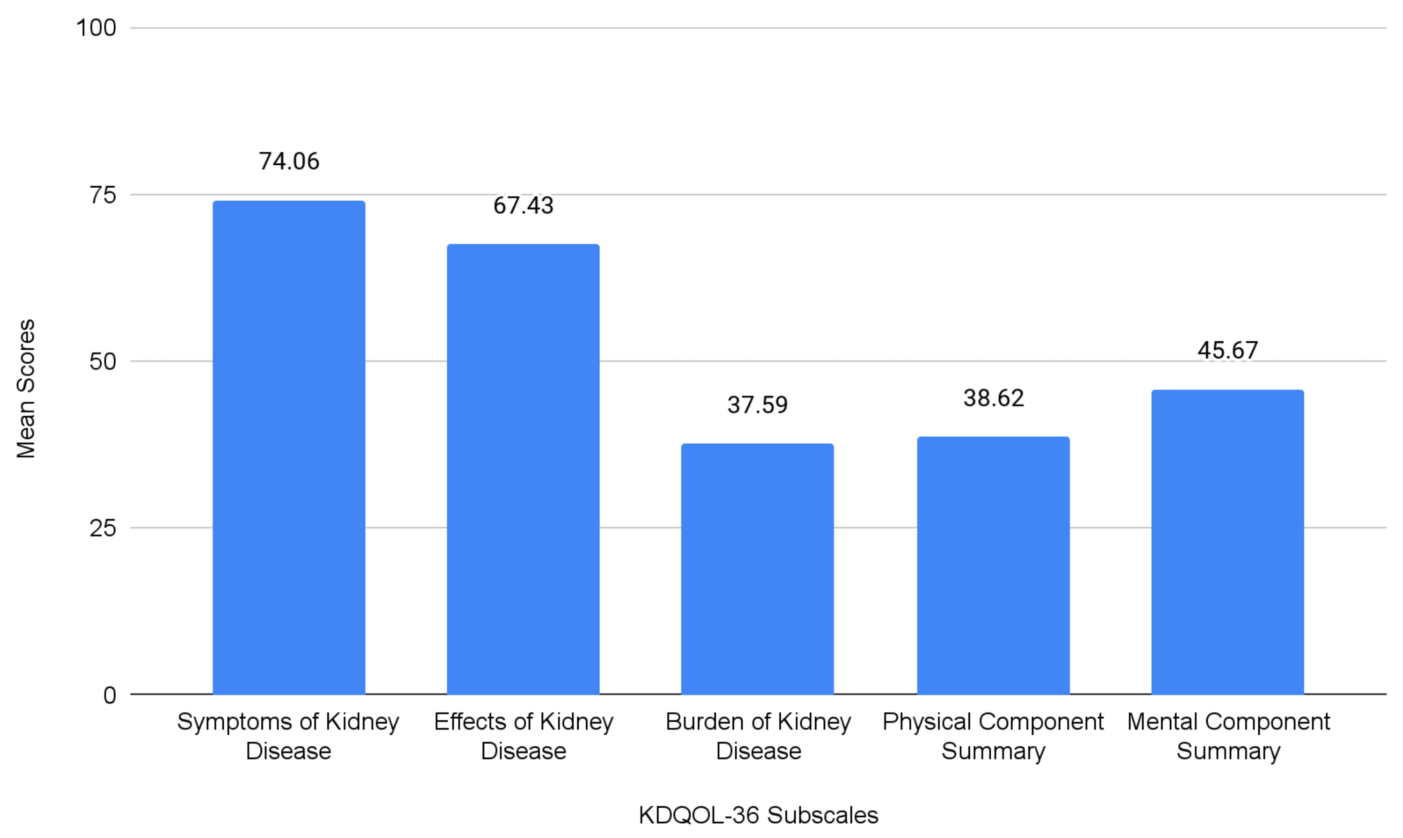
Mean scores of KDQOL-36 subscales Kidney Disease and Quality of Life Instrument (KDQOL-36^TM^) (RAND Corporation, Santa Monica, CA).

QoL results based on the five KDQOL-36™ subscales are displayed in Figure 2. Burden of the Kidney disease, Physical Component Summary (PCS), and Mental Component Summary (MCS) subscales showed the lowest mean scores with 37.59 ± 26.89 (Max: 100, Min:0), 38.62 ± 10.35 (Max: 58.54, Min: 18.30), and 45.67 ± 11.25 (Max: 66.50, Min: 15.43) respectively. The highest mean scores were observed in Symptoms and Problems and Effects of Kidney Disease subscales, with 74.06 ± 18.44 (Max: 100, Min: 0) and 67.43 ± 21.89 (Max: 100, Min: 0), respectively.

Inferential statistics

**Figure 3:**
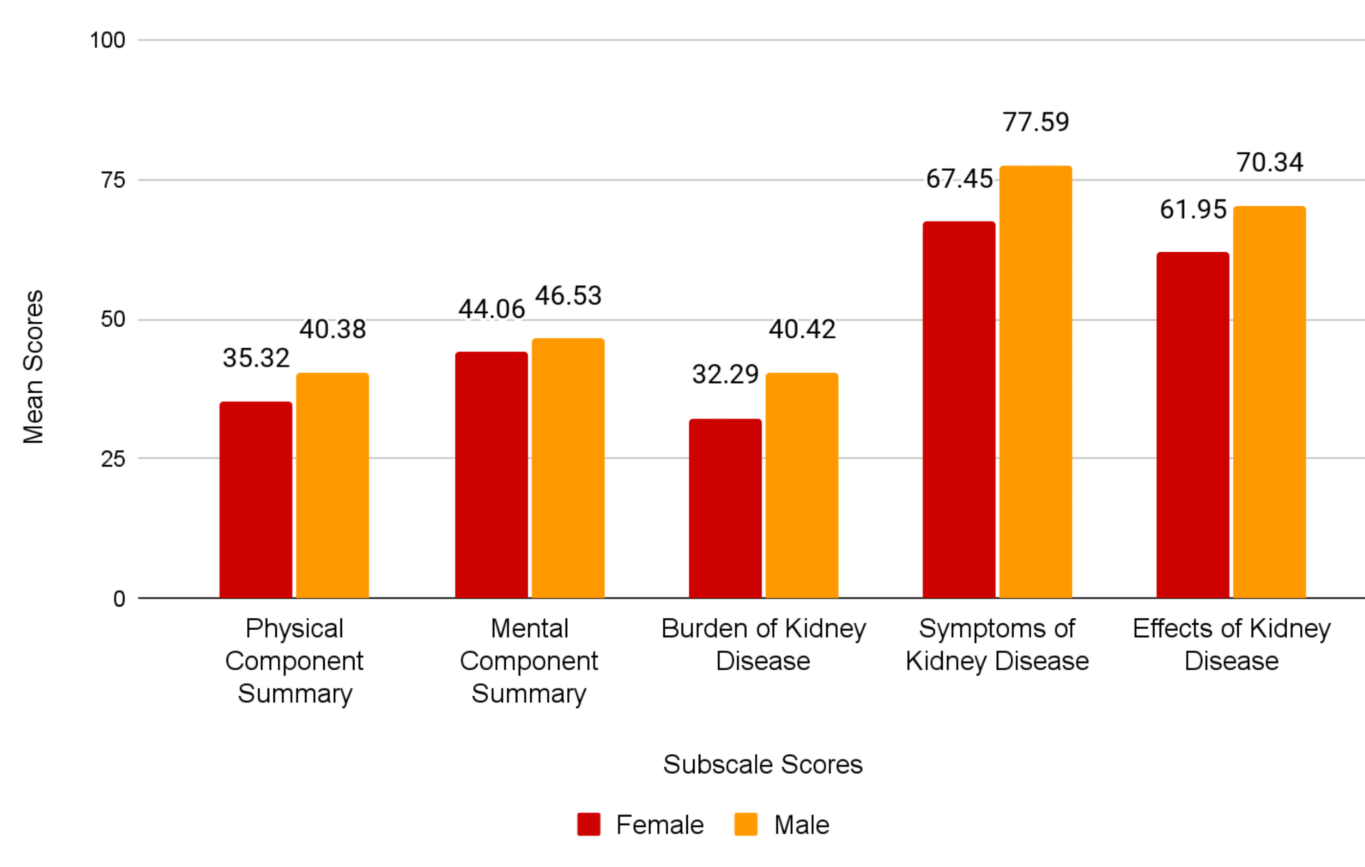
Mean scores of participants based on gender

Females were observed to have lower scores than males in all KDQOL-36™ subscales. However, Mann-Whitney U tests revealed significantly different PCS (p=0.008) and Symptoms/Problems (p=0.003) scores; other scores were not significantly different (p=0.061 for Effects of Kidney Disease, p=0.051 for Burden of Kidney Disease, and p=0.216 for MCS). Mean scores for each subscale are shown in Figure 3 based on the gender of participants. Participants older than 65 had significantly higher scores in the Effects of Kidney Disease and Burden of Kidney Disease subscales (p=<0.001 and p= 0.009, respectively). Associations of age groups with other subscales are shown in Table 4. 

**Table 4:** Associations between participants' age groups and KDQOL-36 subscales ^a^Using Mann-Whitney U Test Kidney Disease and Quality of Life Instrument (KDQOL-36^TM^) (RAND Corporation, Santa Monica, CA).

Associations between participants' age groups and KDQOL-36 subscales.					
	≤ 65 (n=55)		>65(n=83)		
	Mean±SD	Median (Min–Max)	Mean±SD	Median (Min–Max)	p^a^
Symptoms/Problems	71.52±18.85	77.08 (33.33-100.00)	75.75±18.08	79.17 (0.00-100.00)	0.178
Effects of Kidney Disease	58.28±20.56	57.14 (0.00-100.00)	73.49±20.72	78.13 (18.75-100.00)	<0.001
Burden of Kidney Disease	29.55±21.70	25.00 (0.00-87.50)	42.92±28.74	43.75 (0.00-100.00)	0.009
Physical Component Summary	37.10±9.68	36.70 (18.30-57.32)	39.63±10.71	38.58 (19.89-58.54)	0.175
Mental Component Summary	45.32±11.88	45.89 (15.85-65.83)	45.89±10.89	47.21 (15.43- 66.50)	0.823

Table 5 shows the associations between subscale scores and the educational levels of participants. A significant association between patients’ occupational status and Symptoms/Problems subscale was revealed (p<0.001). Associations of occupational status with other subscales are shown in Table 6. A significant association between having a caregiver and the Symptoms/Problems subscale was revealed (p=0.003). Associations between caregivers and other subscales are shown in Table 7.

**Table 5:** Associations between the education level of participants and KDQOL-36 subscales Kidney Disease and Quality of Life Instrument (KDQOL-36^TM^) (RAND Corporation, Santa Monica, CA).

	None (n=10)		Primary Education (n=59)		Secondary Education (n=54)		Tertiary Education (n=15)		p
	Mean±SD	Median (Min–Max)	Mean±SD	Median (Min–Max)	Mean±SD	Median (Min–Max)	Mean±SD	Median (Min–Max)	
Symptoms/ Problems	62.08±26.27	69.79 (0.00-89.58)	73.83±16.56	77.08 (33.33-100.00)	75.89±19.63	82.29 (33.33-100.00)	76.39±12.93	79.17 (56.25-97.92)	0.276
Effects of Kidney Disease	55.05±30.16	62.50 (0.00-100.00)	67.81±19.23	68.75 (28.13-100.00)	67.88±22.27	70.32 (25.00-100.00)	72.53±23.62	75.00 (31.25-100.00)	0.463
Burden of Kidney Disease	44.38±30.68	43.75 (0.00-93.75)	34.85±25.06	31.25 (0.00-100.00)	37.62±27.75	34.38 (0.00-100.00)	43.75±29.12	50.00 (0.00-93.75)	0.629
Physical Component Summary	39.65±9.98	39.93 (19.89-53.07)	38.02±10.51	36.81 (18.30-58.54)	37.99±9.97	37.75 (22.78-55.78)	42.56±11.41	42.21 (23.86-57.32)	0.558
Mental Component Summary	41.29±11.75	42.37 (15.85-58.58)	45.63±11.43	46.39 (15.43-65.83)	46.27±11.31	46.96 (20.16-66.50)	46.54±10.45	46.75 (22.46-61.51)	0.583

**Table 6:** Associations between participants' occupational status and KDQOL-36 subscales Kidney Disease and Quality of Life Instrument (KDQOL-36^TM^) (RAND Corporation, Santa Monica, CA).

	Not working (n=40)		Working (n=9)		Retired Forced (n=31)		Retired Preferred (n=58)		p
	Mean±SD	Median (Min–Max)	Mean±SD	Median (Min–Max)	Mean±SD	Median (Min–Max)	Mean±SD	Median (Min–Max)	
Symptoms/Problems	63.39±19.03	60.42 (0.00-91.67)	76.16±20.68	79.17 (33.33-100.00)	77.15±13.60	79.17 (43.75-100.00)	79.45±17.23	83.33 (33.33-100.00)	<0.001
Effects of Kidney Disease	60.44±23.36	62.50 (0.00-100.00)	60.22±19.80	56.25 (34.38-85.71)	69.21±18.99	71.88 (31.25-96.88)	72.41±21.55	78.13 (28.13-100.00)	0.045
Burden of Kidney Disease	34.38±26.25	28.13 (0.00-100.00)	41.67±21.88	50.00 (12.50-81.25)	32.06±24.78	31.25 (0.00-93.75)	42.13±28.79	40.63 (0.00-100.00)	0.259
Physical Component Summary	35.75±10.23	34.59 (18.30-55.17)	41.68±10.43	42.21 (27.45-57.32)	36.90±8.33	36.81 (22.66-53.88)	41.05±10.92	39.19 (22.78-58.54)	0.07
Mental Component Summary	43.75±13.24	45.86 (15.43-65.36)	47.72±9.48	50.48 (26.07-58.83)	46.07±8.93	46.22 (28.60-61.51)	46.45±11.22	47.37 (17.07-66.50)	0.716

**Table 7:** Associations between participants' caregivers and KDQOL-36 subscales Kidney Disease and Quality of Life Instrument (KDQOL-36^TM^) (RAND Corporation, Santa Monica, CA).

	Spouse (n=71)		Son/Daughter (n=36)		Sibling (n=8)		Others (n=13)		None (n=10)		p
	Mean±SD	Median (Min–Max)	Mean±SD	Median (Min–Max)	Mean±SD	Median (Min–Max)	Mean±SD	Median (Min–Max)	Mean±SD	Median (Min–Max)	
Symptoms/Problems	80.28±14.40	83.33 (41.67-100.00)	65.86±23.56	72.92 (0.00-100.00)	75.26±12.77	72.92 (60.42-95.83)	66.83±14.72	62.50 (47.92-93.75)	67.92±17.18	65.63 (41.67-87.50)	0.003
Effects of Kidney Disease	70.53±19.59	71.88 (31.25-100.00)	63.38±24.85	70.32 (0.00-100.00)	73.27±16.93	73.00 (46.43-100.00)	65.73±23.27	65.63 (31.25-100.00)	57.50±26.11	50.00 (28.13-100.00)	0.418
Burden of Kidney Disease	40.14±27.80	37.50 (0.00-100.00)	32.12±22.47	25.00 (0.00-93.75)	42.19±30.57	34.38 (6.25-87.50)	35.10±30.46	25.00 (0.00-93.75)	38.75±29.43	40.63 (0.00-100.00)	0.685
Physical Component Summary	40.88±10.50	41.82 (19.34-58.54)	35.83±10.61	33.07 (19.89-55.72)	36.63±10.49	36.51 (18.30-53.07)	34.59±7.18	36.37 (25.15-53.19)	39.48±9.03	38.52 (26.81-56.99)	0.066
Mental Component Summary	46.91±9.16	46.75 (22.46-66.50)	45.38±11.93	46.45 (17.07-61.41)	42.17±12.57	43.79 (26.07-62.76)	45.00±15.07	48.55 (15.85-65.36)	41.56±15.90	46.02 (15.43-58.80)	0.84

Associations between patients’ comorbidities and their KDQOL-36™ scores were further investigated using the Mann-Whitney U Test. Associations of having hypertension, diabetes, cardiovascular diseases, and musculoskeletal diseases with the different subscale mean scores of KDQOL-36™ are shown in Tables 8, 9, 10, 11, respectively. Tests revealed that having either hypertension (p=0.049) or musculoskeletal diseases (p=0.002) as comorbidities were significantly associated with lower Symptoms/Problems subscale scores.

**Table 8:** Association between hypertension as a comorbidity and KDQOL-36 subscales Kidney Disease and Quality of Life Instrument (KDQOL-36^TM^) (RAND Corporation, Santa Monica, CA).

Subscales	Hypertension	Mean±SD	Median (Min-Max)	p
Symptoms/Problems	Yes	71.00±19.67	77.08 (0.00-100.00)	0.049
	No	77.60±16.35	82.29 (35.42-100.00)	
Effects of Kidney Disease	Yes	67.64±22.88	71.88 (18.75-100.00)	0.819
	No	67.18±20.88	68.75 (0.00-100.00)	
Burden of Kidney Disease	Yes	37.16±26.95	31.25 (0.00-100.00)	0.812
	No	38.09±27.02	37.50 (0.00-100.00)	
Physical Component Summary	Yes	38,85±10,83	37.47 (19.34-58.54)	0.828
	No	38.36±9.85	37.81 (18.30-57.32)	
Mental Component Summary	Yes	45.63±11.76	47.26 (15.43-65.83)	0.819
	No	45.71±10.73	46.22 (22.46-66.50)	

**Table 9:** Association between diabetes mellitus as a comorbidity and KDQOL-36 subscales Kidney Disease and Quality of Life Instrument (KDQOL-36^TM^) (RAND Corporation, Santa Monica, CA).

Subscales	Diabetes	Mean±SD	Median (Min-Max)	p
Symptoms/Problems	Yes	71.00±19.67	77.08 (0.00-100.00)	0.895
	No	77.60±16.35	82.29 (35.42-100.00)	
Effects of Kidney Disease	Yes	67.64±22.88	71.88 (18.75-100.00)	0.141
	No	67.18±20.88	68.75 (0.00-100.00)	
Burden of Kidney Disease	Yes	37.16±26.95	31.25 (0.00-100.00)	0.193
	No	38.09±27.02	37.50 (0.00-100.00)	
Physical Component Summary	Yes	38.85±10.83	37.47 (19.34-58.54)	0.165
	No	38.36±9.85	37.81 (18.30-57.32)	
Mental Component Summary	Yes	44.46±12.37	45.30 (15.43-65.83)	0.421
	No	46.40±10.53	46.63 (22.46-66.50)	

**Table 10:** Association between cardiovascular diseases as comorbidities and KDQOL-36 subscales Kidney Disease and Quality of Life Instrument (KDQOL-36^TM^) (RAND Corporation, Santa Monica, CA).

Subscales	Cardiovascular	Mean±SD	Median (Min-Max)	p
Symptoms/Problems	Yes	71.00±19.67	77.08 (0.00-100.00)	0.169
	No	75.34±18.14	79.17 (0.00-100.00)	
Effects of Kidney Disease	Yes	67.64±22.88	71.88 (18.75-100.00)	0.14
	No	69.90±21.48	71.88 (0.00-100.00)	
Burden of Kidney Disease	Yes	37.16±26.95	31.25 (0.00-100.00)	0.364
	No	38.41±26.14	37.50 (0.00-100.00)	
Physical Component Summary	Yes	38.85±10.83	37.47 (19.34-58.54)	0.178
	No	39.34±10.61	38.28 (18.30-58.54)	
Mental Component Summary	Yes	45.63±11.76	47.26 (15.43-65.83)	0.065
	No	46.85±10.21	47.21 (15.85-65.36)	

**Table 11:** Association between musculoskeletal diseases as comorbidities and KDQOL-36 subscales Kidney Disease and Quality of Life Instrument (KDQOL-36^TM^) (RAND Corporation, Santa Monica, CA).

Subscales	Musculoskeletal	Mean ± SD	Median (Minimum-Maximum)	p
Symptoms/Problems	Yes	61.06±13.32	62.50 (37.50-83.33)	0.002
	No	75.42±18.41	79.17 (0.00-100.00)	
Effects of Kidney Disease	Yes	64.42±16.85	65.63 (31.25-87.50)	0.466
	No	67.74±22.38	71.88 (0.00-100.00)	
Burden of Kidney Disease	Yes	38.46±16.51	37.50 (12.50-62.50)	0.573
	No	37.50±27.79	31.25 (0.00-100.00)	
Physical Component Summary	Yes	34.36±7.40	33.71 (22.66-49.24)	0.129
	No	39.06±10.53	38.23 (18.30-58.54)	
Mental Component Summary	Yes	44.79±11.26	45.89 (25.73-61.08)	0.76
	No	45.76±11.29	46.75 (15.43-66.50)	

Table 12 shows the correlation between sociodemographic characteristics, laboratory parameters, and quality of life assessed through KDQOL-36™. Significant positive correlations were found between age and all three kidney disease-specific subscale scores, namely, Symptoms/Problems of kidney disease (p=0.045), Effects of Kidney Disease (p<0.001), and Burden of Kidney Disease (p=0.009). Alkaline phosphatase (ALP) showed negative correlations with Symptoms/Problems of kidney disease and PCS subscales (p<0.001 and p=0.001, respectively). Similarly, the number of comorbidities and C-reactive protein (CRP) showed negative correlations with the Symptoms/Problems of Kidney Disease subscale (p=0.002 and p=0.016, respectively). CRP also showed a negative correlation with MCS subscale (p=0.004). Alanine transaminase (ALT) had a positive correlation with Effects subscale (p=0.034).

**Table 12:** Correlation between sociodemographic and laboratory data and KDQOL-36 subscales KDQOL-36^TM^: Kidney Disease and Quality of Life Instrument (RAND Corporation, Santa Monica, CA); r: Spearman’s Correlation coefficient; CK: Creatinine Kinase; ALP: Alkaline Phosphatase; PTH: Parathormone; AST: Aspartate Transaminase; ALT: Alanine Transaminase; CRP: C-reactive protein; WBC: white blood cells; Hb: Hemoglobin; Ht: Hematocrit; MCV: Mean corpuscular volume. Plt: platelets. * Correlation is significant at the 0.05 level (2-tailed); ** Correlation is significant at the 0.01 level (2-tailed).

KDQOL-36™										
Variables	Symptoms and Problems		Effects of the Kidney Disease		Burden of the Kidney Disease		Physical Component Summary		Mental Component Summary	
	r	p	r	p	r	p	r	p	r	p
Age	0.171*	0.045	0.370**	<0.001	0.222**	0.009	0.088	0.303	0.109	0.203
Months Since	-0.074	0.404	-0.01	0.912	-0.017	0.848	-0.133	0.134	0.043	0.631
Comorbidities	-0.259**	0.002	-0.024	0.777	0.029	0.74	-0.09	0.296	-0.081	0.345
CK	0.001	0.994	-0.125	0.149	-0.008	0.931	0.107	0.219	0.026	0.769
ALP	-0.296**	<0.001	-0.131	0.129	-0.165	0.056	-0.277**	0.001	-0.135	0.119
PTH	-0.134	0.143	-0.021	0.823	-0.056	0.539	-0.027	0.772	-0.075	0.412
Total Protein	-0.045	0.601	-0.126	0.146	-0.093	0.284	-0.043	0.622	-0.167	0.053
Albumin	-0.011	0.898	-0.045	0.599	0.03	0.727	0.023	0.786	0.01	0.904
AST	-0.063	0.47	0.027	0.759	-0.003	0.975	-0.114	0.186	-0.065	0.455
ALT	0.068	0.434	0.182*	0.034	0.046	0.591	0.016	0.852	0.052	0.547
CRP	-0.220*	0.016	-0.122	0.183	-0.097	0.29	-0.143	0.116	-0.260**	0.004
WBC	-0.029	0.733	-0.054	0.533	0.016	0.85	0.021	0.809	-0.083	0.332
Hb	0.052	0.548	0.015	0.859	0.032	0.709	0.038	0.656	0.136	0.114
Ht	-0.013	0.88	-0.031	0.715	-0.037	0.664	-0.089	0.301	0.145	0.091
MCV	-0.003	0.976	0.09	0.292	0.059	0.492	0.035	0.681	0.057	0.509
Plt	-0.046	0.597	-0.12	0.163	-0.116	0.178	-0.008	0.924	-0.075	0.386

In order to assess the significant predictors of KD-QoL^TM^ subscales, Multiple Linear Regression analyses using the ‘stepwise’ method were conducted. From sociodemographic variables, gender (p=0.006), number of comorbidities (p=0.005), and caregiver (p=0.009) were found to be significant predictors, whereas, among laboratory parameters, total protein (p=0.030), C-reactive protein (p=0.032) and hemoglobin (p=0.012) were found to be significant predictors. The number of comorbidities a patient had was found to be the strongest predictor, whereas the C-reactive protein was found to be the weakest predictor of KD-QoL^TM ^subscales. Detailed results of the regression analyses are shown in Table 13.

**Table 13:** Results of multiple linear regression analyses ^a^Predictors of Symptoms/Problems of Kidney Disease subscale; ^b^Predictors of Physical Component Summary subscale; ^c^Predictors of Mental Component Summary; ^d^Predictors of Burden of Kidney Disease subscale.

	B	SE B	β	t	p
Gender ^a^	8.494	1.158	-0.240	-2.860	0.006
Number of comorbidities ^a^	-3.313	3.016	0.237	2.817	0.005
Caregiver ^b^	-1.927	0.721	-0.239	-2.672	0.009
Total Protein (g/dl) ^c^	-3.895	1.768	-0.206	-2.203	0.030
C-reactive protein (mg/L) ^d^	-2.039	0.936	-0.207	-2.178	0.032
Hemoglobin (g/dl) ^c^	1.616	0.633	0.238	2.554	0.012

## Discussion

Long-term HD in ESRD patients has detrimental effects on HR-QoL. Several studies demonstrate poor HR-QoL of these patients with respect to the general population despite ongoing developments in treatment options [11]. Numerous aspects of these patients’ lives are negatively affected, ranging from the inability to attend social activities, to marital problems, to financial difficulties, and feelings of depression [12]. It is, therefore, vital that the HR-QoL of these patients be monitored and the factors adversely affecting it be identified to better understand the patient’s condition. This can help direct interventions to subgroups that have been pinpointed to have comparatively poor perceived health [11]. The dominant therapeutic objective in these patients is to allow them to fully enjoy life by bettering their functional capabilities [13].

A study conducted at Alexandria University in Egypt showed that women had lower HR-QoL scores compared to men. They state that psychosocial factors play an important role in this discrepancy [14]. These results are consistent with the results of the present study. Statistically significant differences were found, especially among the Symptoms/Problems of Kidney Disease and PCS subscale scores of KDQOL-36^TM^. Males were found to better tolerate the symptoms of their kidney disease, as well as to be physically better off.

Another study from Greece found that participants with higher education had better HR-QoL scores [15]. Naturally, we would expect these patients to have a better understanding of their disease and, therefore, be more compliant with their treatment regimens. Similarly, higher income would be expected in more educated patients and, as a result, the ability to afford superior treatment modalities. The results of the present study are inconsistent with those of previous studies that identified differences in quality of life based on education levels, in that tertiary education level graduate patients had similar scores to those who graduated from only Primary or Secondary levels of education.

When the HR-QoL of patients in two separate dialysis units in India was assessed, they found employment to be a vital factor in improving the HR-QoL. It was discovered that the retired group scored the lowest [13]. These results are contradictory to the findings of the present study, which observed retired patients (those either forced into retirement by their kidney disease or those that intentionally preferred retirement) to score the highest among those who were employed and those who were unemployed. Interestingly, unemployed patients were found to have the lowest scores. We can speculate that retired patients in Northern Cyprus see retirement as an opportunity to enjoy life, as opposed to a handicap. They feel more free and independent, find more time to spend with their families, and pursue their hobbies of interest, ultimately yielding better quality of life scores.

Re-visiting the study conducted in Greece, we find that they also demonstrate a correlation between age and HR-QoL. They divided participants into three groups according to age. Those above the age of 60 years were found to have the worst HR-QoL scores, followed by the 41-60 years age group, and finally, those below the age of 40 [15]. There is a visible trend that depicts the decline of HR-QoL as age increases. These results differ from the results of the present study, which found patients above the age of 65 to have the highest HR-QoL scores. Statistically significant improvements in scores were observed in the Effects of kidney disease and the Burden of kidney disease subscales of KDQOL-36^TM^ of elderly participants. We relate the cause of this discrepancy to survivorship bias, a type of selection bias. We were led to focus on only the survivors while overlooking the patients who had passed away.

The results of the present study are consistent with a Korean study that demonstrated the worsening of HR-QoL as the number of comorbidities increased. They found diabetes mellitus (39.7%) to be the most common comorbidity, followed by hypertension (29.1%) [16]. Patients with multiple comorbidities in the current study were found to have significantly lower scores in the Symptoms/problems of Kidney Disease subscale of KDQOL-36^TM^. Similarly, diabetes mellitus (38.1%) and hypertension (54%) were also found to be the two most common comorbidities. 

Scores of different countries from separate studies were compared with those of the present study in Northern Cyprus to reveal visible trends (Table 14). Generally speaking, the United States [17] demonstrated higher scores in MCS, Symptoms/Problems of Kidney Disease, Effects of Kidney Disease, and Burden of Kidney Disease subscales of KDQOL-36^TM^. The lowest scores belong to a city in India named Kerala [18]. Surprisingly, Northern Cyprus out-scored European countries [8] in all domains of HR-QoL. We speculate that India had the lowest scores as it is still a developing country where poverty is prevalent. One would expect that they would have less advanced healthcare opportunities. In contrast, the United States is a developed country with a higher GDP. Naturally, they would be expected to have more advanced healthcare facilities and, as a result, overall better HR-QoL scores in their patients.

**Table 14:** KDQOL-36 scores according to country Kidney Disease and Quality of Life Instrument (KDQOL-36TM) (RAND Corporation, Santa Monica, CA).

	PCS	MCS	Symptoms	Effects	Burden
India (Nayana et al., 2016) [18]	36.49	41.83	68.69	46.32	33.50
Europe (Fukuhara et al., 2003) [8]	34.70	44.10	70.40	57.90	36.80
Japan (Fukuhara et al., 2003) [8]	40.00	44.00	73.80	66.70	27.60
Present Study	38.62	45.67	74.06	67.43	37.59
United States (Hall et al., 2018) [17]	34.50	50.90	78.50	74.30	52.60

Limitations

We acknowledge that the effectiveness of any interventions, had they been made, on these patients could not be followed up within the scope of this cross-sectional study. A cohort study would have to be conducted in order to test the effectiveness of interventions. However, our study being cross-sectional in its nature does not diminish its predictive potential of mortality, as recognized by a study that states that the “most recent measure of quality of life is more predictive of mortality than the change in quality of life over time” [17].

## Conclusions

This study has shown that multiple aspects of health-related quality of life (HR-QoL) of patients receiving hemodialysis in governmental hospitals in Northern Cyprus were markedly impaired. Precisely, Burden of the Kidney disease, Physical Component Summary, and Mental Component Summary subscales showed the lowest KDQoL-36™ mean scores. Furthermore, being a female, having multiple comorbidities, and being currently unemployed were all factors found to be independently associated with patients having lower KDQoL-36™ mean scores and subsequently impaired HR-QoL.

Taking everything into account, the integration of HR-QoL surveillance in patients receiving hemodialysis may have an advantageous therapeutic effect through early identification of risk factors and, as a result, allowing a more targeted and patient-centered approach. We urge healthcare providers to be more cognizant of their patients’ HR-QoL and its evidence-based impact on disease course by making it part of routine care. Additionally, it is crucial for them to be mindful of both the positive and negative factors associated with their patients’ mean scores. Nevertheless, further studies regarding the implementation of routine HR-QoL surveillance and targeted interventions are still required to truly understand their potential therapeutic benefits.
